# Evidence on global public health cancer strategies

**DOI:** 10.1186/s44263-025-00197-z

**Published:** 2025-08-20

**Authors:** Ben Cranfield, Tanimola Martins, Suzanne Scott

**Affiliations:** 1 BioMed Central, Springer Nature, London, UK; 2 University of Exeter Collaboration for Academic Primary Care (APEx), University of Exeter Medical School, Exeter, EX1 2LU UK; 3 Wolfson Institute of Population Health, Queen Mary University of London, London, UK

*Cancer remains a significant global public health concern. The challenge of reducing global mortality rates for cancer requires a holistic approach, drawing from expertise from communities world-wide.*

Variation in global cancer mortality rates are an indication of continuing health inequity. Our collection on ‘Implementation of cancer strategies in primary care: Addressing global disparities’ (https://link.springer.com/collections/bihfdhbege) aimed to provide a platform to distribute evidence on cancer strategies. The content within the collection is a timely reminder of the pioneering work that helps to generate context-specific cancer strategies for reducing inequities in cancer care.

The primary health care setting is, for most people, where opportunities for health care provision starts. However, the timeliness of presentation to a medical practitioner is a key factor that can determine health outcomes—for suspected cancer, more timely help-seeking with the healthcare system could increase the chances of earlier cancer diagnosis and subsequently better health outcomes [1]. However, as outlined by Whittaker and colleagues in their perspective article, there are a number of challenges in understanding help-seeking for possible cancer [2]. How help-seeking is measured can introduce inequity if explored as a dichotomous variable (i.e. yes/no) as this does not capture the complexity of the behaviour. The authors express how research into help-seeking behaviour should further explore the interrelationship between sociodemographic characteristics, potential mediators and real-life help-seeking behaviour. Furthermore, obtaining data on help-seeking from routinely collected data can be challenging and given the current underrepresentation and often missing data associated with minority populations may prevent insight into equity. Yet, there is scope for optimism as evident from the availability of large datasets with 100% coverage, which the authors indicate could help to drive improvements in healthcare inequalities.

Screening is a critical step of the cancer diagnostic process. The collection presents data on this from a range of contexts. A cross-sectional study by Adefemi and colleagues revealed that while colorectal cancer (CRC) screening uptake increased across the Atlantic provinces of Canada between 2010 and 2017 (the most recent data for CRC screening in this region), barriers to and disparities in screening participation persist [3]. In particular, disparities existed among populations in their 50’s and in socio-economically disadvantaged groups. In the USA, Luque and colleagues conducted a pragmatic randomised controlled trial to explore the effectiveness of a community health advisor CRC screening educational intervention on stool test completion in an African American primary care patient population [4]. The completion of stool tests after 12 months increased significantly for both groups, yet the intervention group also reported increased CRC knowledge and perceived susceptibility to CRC. The findings highlight an important role that community health centres can play in increasing CRC screening.

The collection also provides evidence to help reduce barriers to cancer screening for women. Breast cancer is the second most commonly diagnosed cancer globally [5], yet screening methods vary significantly between countries. Badal and colleagues argue that risk-based screening is more appropriate than aged-based screening in limited resource settings as this can help to optimise scarce resources by targeting women at highest risk, improving screening efficiency despite resource constraints [6]. Bangladesh represents a setting with limited resources where Nessa and colleagues assessed data from over 1.5 million women to review the cervical cancer surveillance situation based on national electronic registry data [7]. The electronic health system allowed the authors to better understand the current cervical cancer screening situation in Bangladesh, which could support the monitoring, evaluation and management of screen-positive women. The approach was considered to be viable and effective in this context and could have implications for similar settings. Lastly, in a remote region of northwestern Ecuador, researchers explored the prevalence of human papillomavirus (HPV) genotypes in women with different ethnicities, highlighting higher HPV genotype prevalence in this region compared to others and thus opportunities for targeted intervention (such as vaccination programs) [8].

While the studies span a range of contexts, there is further opportunity to focus on innovative or emergent approaches, such as the integration of digital tools, AI-driven triage systems or community-based risk stratification models, especially in low-resource settings. These areas represent an important and evolving frontier in global cancer control, and require investigation to ensure they are contextually appropriate.

The collection contributes towards global ambitions to reduce inequalities in cancer care. At BMC Global and Public Health, we will continue to publish impactful and exciting content that promotes health equity, supporting communities worldwide.

## Data Availability

Not applicable.
